# A Quality Improvement Initiative to Reduce Prescription Error in a Pediatrics Outpatient Department at a Secondary-Level Community Hospital

**DOI:** 10.7759/cureus.56004

**Published:** 2024-03-12

**Authors:** Ayush Gupta, Sumit Malhotra, Suprakash Mandal, Aftab Ahmad, Vasishta Polisetty, Daryavali N Shaik, Ashok K Deorari

**Affiliations:** 1 Department of Neurology, University of Louisville, Louisville, USA; 2 Center for Community Medicine, All India Institute of Medical Sciences, New Delhi, New Delhi, IND; 3 Center for Community Medicine, Teerthanker Mahaveer Medical College and Research Centre, Moradabad, IND; 4 Psychosis Collective, The Francis Crick Institute, London, GBR; 5 Department of Pediatrics, All India Institute of Medical Sciences, New Delhi, New Delhi, IND

**Keywords:** prescription, outpatient, pediatric, quality, error

## Abstract

Background

Medication errors are common, especially by new trainees in primary care settings. Our study aimed at reducing the rate of prescription error in the pediatric outpatient department (OPD) of a secondary healthcare center in suburban north India using a quality improvement methodology.

Methods

Based on a survey and focused group discussion (FGD) involving all stakeholders, the identified problems and difficulties faced during outpatient prescriptions, interventions, and outcome parameters were drafted. The primary outcome measure was the prescription error rate evaluated by a senior resident (SR) of pediatrics, and the secondary outcome measures included the frequency of antibiotic prescriptions and investigations.

Intervention

Two cycles of Plan-Do-Study-Act (PDSA) were conducted on accessible drug formularies and standard treatment protocols for common pediatric conditions.

Results

The mean baseline prescription error was 72.2% (95% confidence interval (CI): 63.2-81.1). After the implementation of the first PDSA cycle, the mean error rate was 46.5% (95% CI: 36.6-56.5). There were eight consecutive points of prescription error below the control limit (63.2% and 81.1%) of the baseline. The PDSA-2 cycle showed the same shift to below the control limit (36.6% and 56.5%). The mean error rate found at the end of the PDSA-2 cycle was 22.5% (95% CI 15.7-29.5). There was no clinically significant difference in the number of investigations or antibiotics prescribed.

Conclusion

The application of standardized drug formularies and standard treatment protocols (STPs) can help reduce prescription errors, especially in a primary care setting. Expansion of such techniques to other centers could be particularly useful.

## Introduction

Medication and prescription errors are common in clinical practice, especially in primary care and resource-limited settings. A medication error is defined as a ‘failure in the treatment process that leads to, or has the potential to lead to, harm to the patient.’ A prescription error, on the other hand, is a kind of medication error defined as a ‘failure in the prescription writing process that results in a wrong instruction about one or more of the normal features of a prescription' [1]. 

Prescription errors result from several key processes, right from the initial evaluation of the patient to the thought process generated in the mind of the treating physician to the expression of that thought in the written language [2,3]. A study reported that 65% of the medication errors were due to prescription errors alone [4]. In a systematic review conducted in 2008, the major types of prescription errors were wrong dosing, wrong frequency, incomplete prescription, wrong drugs, including the prescription of a contraindicated drug, etc. [5].

The Institute of Medicine estimated additional expenses due to preventable medical errors in the United States were approximately $17 billion every year [6]. Medication errors increase morbidity, mortality, and the economic burden on the healthcare system [7]. 

In India, there is a variable proportion of medication and prescription errors based on the level of health setup and type of clinic. A study done among inpatients in the pediatrics ward in Ahmedabad, India, reported a medication error rate of 35%, most of which were prescription errors [4]. Another study conducted in Nagpur, India, revealed the inclusion of antibiotics in 79% of all the pediatric prescriptions assessed [8]. No relevant data for a pediatric population in a secondary hospital could be found from India.

Various factors affecting the occurrence of prescription errors have been identified. Medical staff-related factors include exhaustion due to work pressure and a chaotic work environment [9]. The use of liquid medications, parent measurement of medicines, and low health literacy may further contribute to the occurrence of medication errors [10]. Polypharmacy and a lack of social support have also been identified as patient factors in medication errors by nursing staff [11].

Primary care practitioners usually lack formal training in prescription writing in pediatrics posting in medical colleges. Frequent cycling of trainees in departmental postings or clerkships leaves them with reduced exposure time to a specific specialty. Exposure to the pediatric clinical environment is better correlated to the occurrence of prescription error rather than the number of clinical years or duration of practice [12]. Primary care practitioners and resident doctors primarily treating adults are more prone to a higher frequency of pediatric prescription errors [13].

Various measures to curb the occurrence of prescription errors have been studied to date. Of these, electronic prescriptions have been particularly shown to reduce the occurrence of inpatient medication errors [14-16]. Inculcation of a culture of error reporting, as well as systematic non-punitive approaches, have been shown to decrease errors in clinical settings [17]. It has been shown that structured education programs are effective in reducing errors and sustaining the quality of prescriptions by residents and interns [18,19].

The present study describes a quality improvement initiative to reduce prescription errors in a pediatric outpatient setting of a secondary-level healthcare center in the suburban National Capital Region (NCR) of India. We aimed to reduce the prescription error by 50% of the baseline by two Plan-Do-Study-Act (PDSA) cycles.

## Materials and methods

Study setting

The Comprehensive Rural Health Services Project (CRHSP), Ballabgarh, was a secondary-level sub-district hospital in the Faridabad district of Haryana, on the outskirts of the National Capital Region (NCR). Medical interns were posted in different batches from January to December each year. One pediatric senior resident (SR), one junior resident (JR) of community medicine, and two interns were posted in the pediatric outpatient department (OPD) on a rotation basis. The average doctor-patient ratio is nearly 40 patients per doctor. Sometimes, interns or residents are on leave, or the senior resident doctor gets called to the labor room to attend to childbirth, which further decreases the doctor-patient ratio.

Data collection

**Figure 1:**
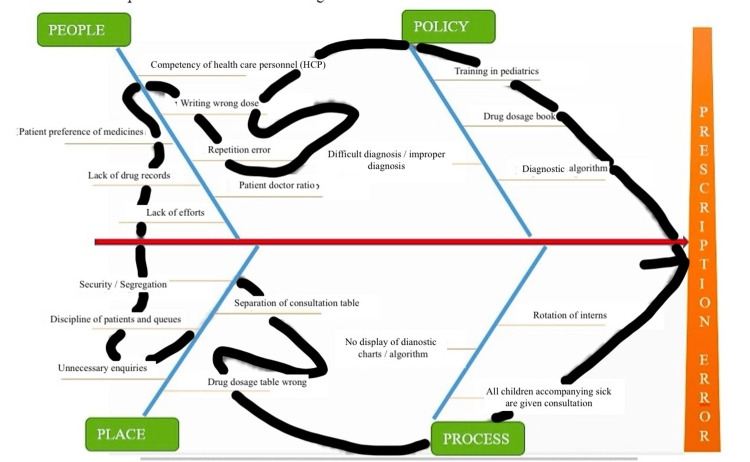
Fishbone analysis for the cause of prescription errors The figure has been created by the authors.

All prescriptions given out by interns or JRs during the first week of December 2018 underwent evaluation. The prescriptions handed out by SR were not included in the study since SR was collecting data and was involved in the analysis of errors. The baseline prescription error was estimated. A focused intervention meeting was held on December 17th, 2018 involving all SR, JR, interns, and staff of pediatric OPD, and the preliminary analysis of prescription errors from the preceding week was discussed. After sensitization of the attendees of the meeting to this problem, the probable reasons were identified and prioritized by fishbone analysis (Figure 1).

The most probable measures to reduce the prescription error were formulated, leading to the initiation of the project. The study lasted for one month. The SR analyzed the errors in prescriptions for the preceding week of follow-up patients using an online form. The online form had fields to record the date of the prescription, the presence of an error, and the type of error in that prescription. The nursing staff ensured the collection of all prescriptions, which were handed out by interns and JR. A small proportion of patients requiring urgent care or referral were seen by the SR during the study period, which was excluded from the study. The nursing staff in the pharmacy was also trained separately for this purpose. A photograph of random prescriptions was also uploaded and discussed in the subsequent quality improvement (QI) meetings. The errors were identified as falling into one of the following categories mentioned in Table 1.

**Table 1:** The operational definitions according to the types of prescription errors

Sl No	Errors	Operational definition
1.	Wrong dose	Prescribing correct medicine for the diagnosis in a dose that is more or less than the recommended dose as per the body weight of the patient
2.	Wrong duration	Prescribing correct medicines for the diagnosis that is less than or more than the recommended duration of treatment
3.	Wrong drug	Prescribing medicine which was not required by the patient, or which is not evidence-based or currently useful for the current diagnosis
4.	Wrong diagnosis	Prescription based on an incorrect diagnosis of the patient
5.	Skipped drug	Prescription which did not contain one or more drugs recommended for treatment or prophylaxis of the patient’s condition

Interventions

A quality improvement tool, the Plan-Do-Study-Act (PDSA) methodology, was adopted in a phasic manner, followed by observation of sustenance. At baseline, the data were collected from December 10 to December 23, 2018 (14 days). The PDSA-1 cycle was run from December 24, 2018 (12 days), intervening by introducing drug formularies. The PDSA-2 cycle was run from January 5 to January 17, 2019 (13 days) with the intervention of the provision of a standard treatment protocol. The data collection for the sustenance phase lasted from January 17 to January 31, 2019 (15 days), with a weekly analysis of the data.

PDSA-1

**Figure 2:**
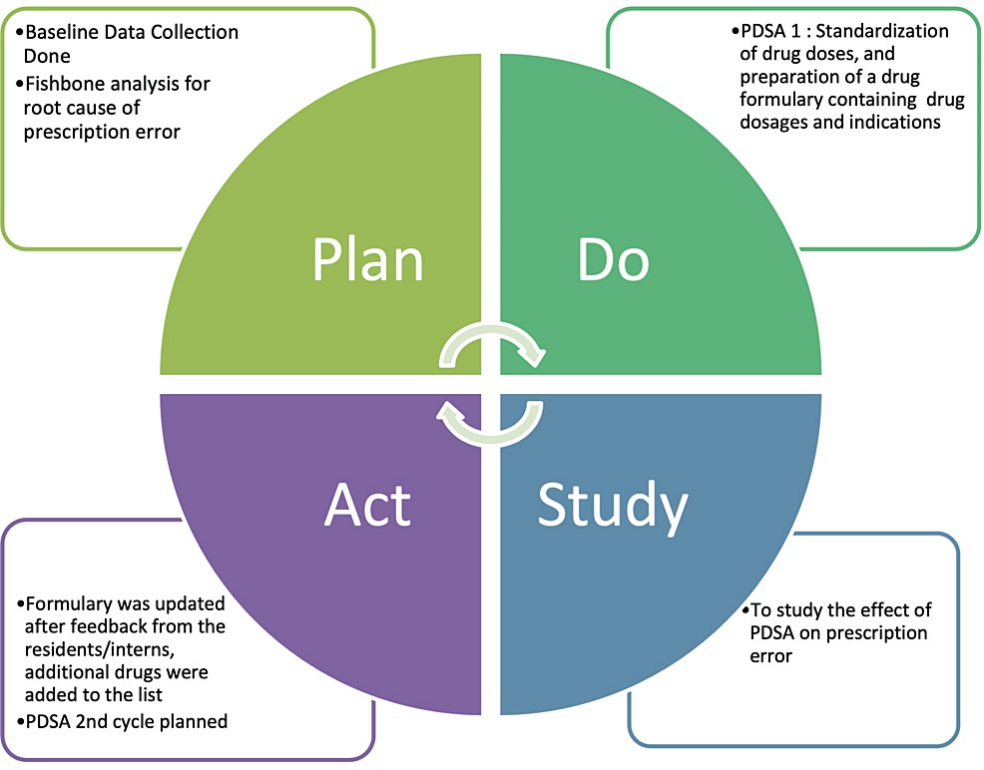
PDSA cycle 1 to accomplish assimilation of the drug formulary The figure has been created by the authors. PDSA: Plan-Do-Study-Act

The first PDSA cycle began with the formation of a QI team consisting of one SR, two JRs, two interns, and one nursing officer. The first cycle targeted a reduction in dosing errors. It was identified that the drug dose chart in OPD had not been updated. So the preparation of new drug formulary for the available and commonly prescribed drugs was planned (Appendix 1). The dosage formulary of the drugs available in the pediatric OPD pharmacy and other commonly prescribed drugs has proper doses and indications. The formulary was further refined after discussion amongst the members of the QI team. From the beginning of the PDSA cycle, feedback was collected, and suitable changes were made to the formulary. A printed copy of the drug formulary was kept on every table in the OPD room to facilitate easy access for consultation. Each day before the start of the OPD, the SR pediatricians briefed the interns and JR about the drug formulary for about ten minutes. This session was intended to accomplish the assimilation of the drug formulary (Figure 2).

PDSA-2

**Figure 3:**
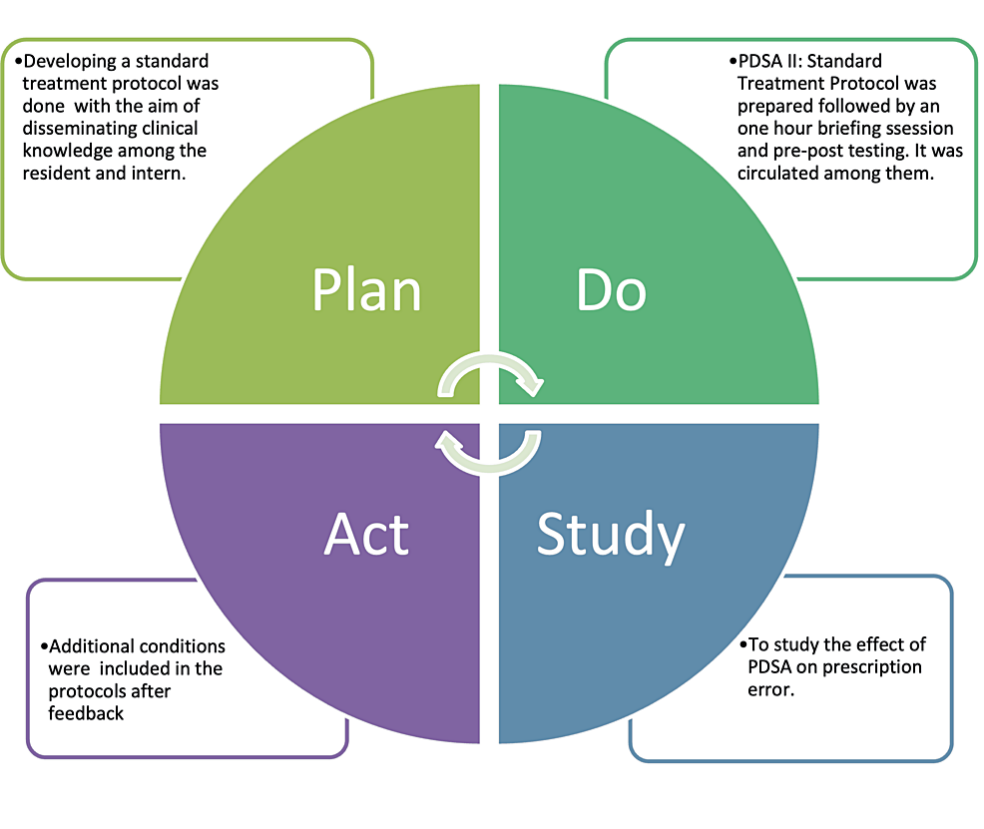
PDSA cycle 2 to disseminate the knowledge by standard treatment protocol (STP) The figure has been created by the authors. PDSA: Plan-Do-Study-Act

In consultation with all QI team members, a standard treatment protocol (STP) was prepared from the standard references and customized for the locally relevant clinical practices and hospital set-up. A briefing session among the resident and intern, along with pre-and post-testing, was conducted, along with recording the feedback. A computerized multiple-choice question (MCQ) based test was taken in pre-and post-session, with the post-test containing five more questions based on clinical knowledge. It contained questions based on common ailments observed in pediatric OPD, their diagnosis, and management, with a maximum score of 20. It was arbitrarily decided that those securing less than 50% marks would be considered to be without proper training and to be subjected to another briefing session. All the interns and JR, but one, were found to secure >50% scores with a mean score of 13 out of 20 after the protocol was taught. The dissemination of knowledge was further enforced by circulating the STP to all the interns and residents. A retrospective collection of data on clinical investigations was also collected at the end of the second PDSA and compared with the help of diagrams (Figure 3).

Post-PDSA-2 survey

An online survey was conducted among all interns and JRs to evaluate their perceptions of the improvement in their confidence level in providing healthcare to pediatric patients after the two rounds of PDSA. Data was collected on a Likert scale of 1 to 10.

Data analysis

Data was entered in a run chart format daily and shared amongst the QI team members at each team meeting. Prescription error was calculated by the ratio of the number of prescriptions with errors per day divided by the number of prescriptions audited, expressed as a percentage.

Statistical significance was analyzed using appropriate statistical tools. Analysis of variance (ANOVA) was used to detect the significance of the difference in mean between errors obtained at baseline, before, and after the first PDSA cycles. Similarly, antibiotic prescriptions were also analyzed.

We also assessed the reduction in prescriptions for oral antibiotics as a ratio of total prescriptions for the day. The pre-post difference amongst various investigations was collected every week and analyzed. A survey for improvement in the confidence levels of the trainees was also done at the end of PDSA-2. The number of lab investigations and patterns of antibiotic prescriptions were expressed in proportion by dividing by the number of patients attending the pediatric OPD, along with estimating the change in pre-and post-intervention.

## Results

The mean baseline prescription error was 72.2% (95% confidence interval (CI): 63.2-81.1). At the end of the PDSA-1 cycle, the mean error rate was 46.5% (95% CI: 36.6-56.5). There were eight consecutive points of prescription error below the control limit (63.2% and 81.1%) of the baseline. The PDSA-2 cycle started considering 46.5% as a baseline. It showed the same decline pattern, keeping the persistence effect below the 95% CI (36.6% and 56.5%). The mean error rate at the end of the PDSA-2 cycle was 22.5% (95% CI 15.7-29.5). The number of prescription errors decreased significantly with a p-value of <0.001 (Table 2).

**Table 2:** Outcome parameters and their measure of significance according to the study phase The proportion of prescription error data has been presented by mean and 95% confidence interval. Statistical significance was calculated by ANOVA. The values are presented as proportions and the n were 390, 434, and 172 at baseline, 1st PDSA, and 2nd PDSA respectively for the secondary parameters. PDSA: Plan-Do-Study-Act; CBC: complete blood count; CI: confidence interval; ANOVA: analysis of variance

Type of parameter	Outcome parameter	Mean value (95% CI)			p-value
		Baseline (n = 390)	PDSA-1 (n = 434)	PDSA-2 (n = 172)	
Primary outcome parameter	Mean prescription error	72.2 (63.2 - 81.1)	46.5 (36.6 - 56.5)	22.5 (15.7 - 29.5)	<0.001
Secondary outcome parameter	Antibiotic prescription	26.4 (15.1-37.7)	27.4 (8.5-46.3)	23.0 (15.2-30.8)	0.85
	Prescription of investigations (%)				
	CBC*	4.1	3.8	3.6	
	Widal*	1.5	0.95	0.55	-
	Organ function tests*	2.8	2.8	2.7	-
	Sputum tests*	0.67	0.68	0.29	-
	X-rays*	4.4	4.0	4.9	-

Weekly data after the PDSA-2 cycle revealed a sustained level of low prescription error. Prescription errors were found to have the same error percentage as in the PDSA-2 cycle at sustenance at two weeks.

**Figure 4:**
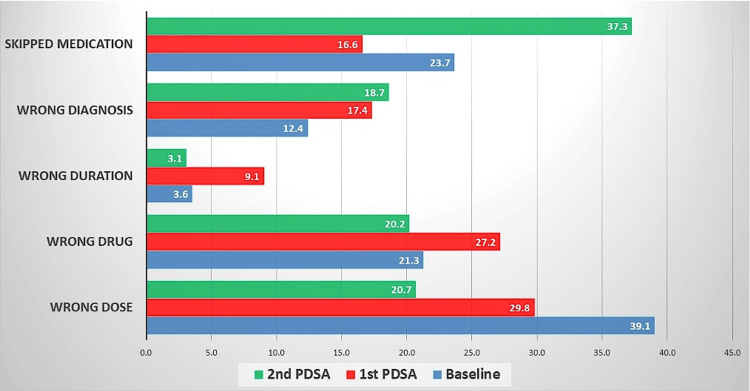
Proportions of types of prescription error at different times of QI intervention The values are presented as proportions and the n were 390, 434, and 172 at baseline, 1st PDSA, and 2nd PDSA respectively. QI: quality improvement; PDSA: Plan-Do-Study-Act

The pattern of prescription errors changed during the course of the study. At the baseline, the wrong dosage accounted for the majority of the prescription errors (39.1%). After the PDSA-1 cycle, there was a gradual decline in the wrong dose domain. At the end of the PDSA-2 cycle, the other four domains, like wrong diagnosis, wrong drug, wrong duration, and skipped medication, also started to decline, though now the skipped medications have become a major contributor to prescription error (37.3%) (Figure 4).

**Figure 5:**
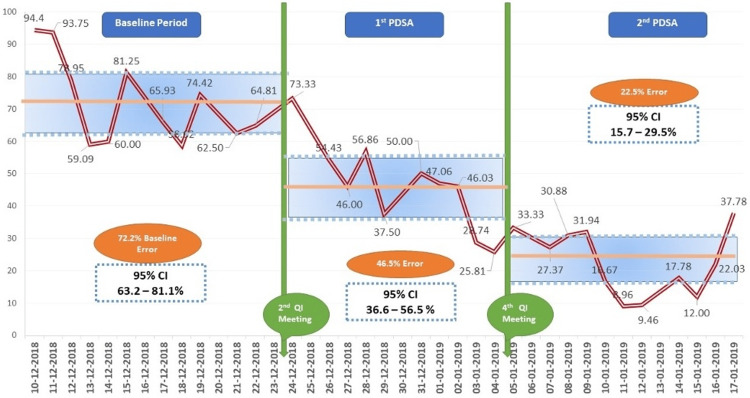
Run chart showing prescription error trends through baseline and PDSA cycles The values are presented as proportions and the n were 390, 434, and 172 at baseline, 1st PDSA, and 2nd PDSA respectively. PDSA: Plan-Do-Study-Act; QI: quality improvement; CI: confidence interval

In the baseline, the prescription error was 72.2% (95% CI 63.2%-81.1%), during the first PDSA, it was 46.5% (95% CI 36.6%-56.5%), and during the second PDSA, it was 23.0% (95% CI 15.2%-30.8%) (Figure 5). 

**Figure 6:**
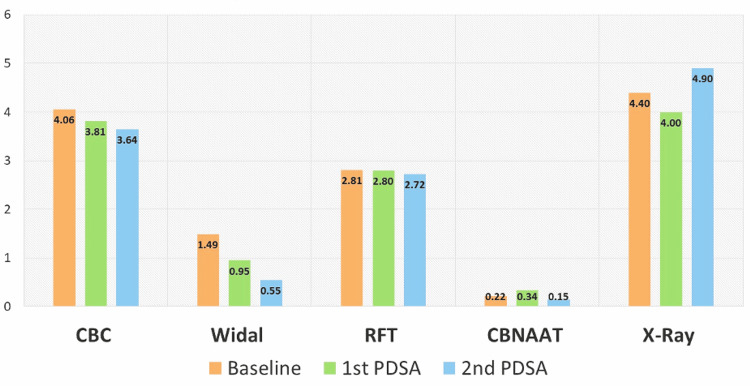
Proportions of the prescriptions of different lab investigations The values are presented as proportions and the n were 390, 434, and 172 at baseline, 1st PDSA, and 2nd PDSA respectively. CBC: complete blood count; RFT: renal function test; CBNAAT: cartridge-based nucleic acid amplification test; PDSA: Plan-Do-Study-Act

Amongst other secondary outcome measures, in terms of investigations, there was no statistically significant difference in the percentage of patients sent for complete blood counts, organ function tests, or X-rays, although testing for the Widal test decreased by 63% (1.5% to 0.5%). We did not analyze the difference statistically due to fewer data points (as data was collected retrospectively on a week-to-week basis) and an apparent lack of clinical significance (Figure 6).

A survey on the perception of improvement in confidence in providing healthcare to pediatric patients was done among all JRs and interns. All 11 interns and JRs who attended the clinics participated in the survey. There was an improvement in the confidence level of providing services to pediatric patients from 5/10 at baseline to 8/10 after two PDSA cycles. There was no difference in responses between interns and residents. A summary of responses is attached in Table 3.

**Table 3:** Secondary outcome parameters (survey results) QI: quality improvement; OPD: outpatient department

Questions asked	Mean score scale (1-10)	Mean score in interns	Mean in score JR
Ease of using drug dosage formulary	8.81	8.87	8.66
Ease of using standard treatment protocol	8.27	8.37	8
Patient improvement in pediatric OPD compared to other OPDs’	8.54	8.62	8.33
Confidence in diagnosis and treatment of pediatric cases "before QI training"	5.09	4.62	6.33
Confidence in diagnosis and treatment of pediatric cases "after QI training"	8.45	8.25	9

## Discussion

This quality improvement study was done at a sub-district hospital in the Faridabad district of Haryana, India. Through the use of two PDSA cycles targeting knowledge acquisition by those who were not traditionally mentored in pediatric medicine, we were successfully able to reduce the prescription error from our baseline mean value of 72.2% (95% CI: 63.2%-81.1%) to 22.5% (95% CI: 15.7%-29.5%) by the end of PDSA-2, which was both clinically and statistically significant (p<0.001). We used the difference between the antibiotic prescription rate before and after the PDSA cycles as our secondary objective, which could serve as a balancing outcome. There was no significant difference in the prescription of commonly used antibiotics between baseline and PDSA cycle 2 (p=0.85). This could happen if we assume that antibiotics were correctly prescribed beforehand and were continually prescribed during these PDSA cycles. However, the number of prescriptions containing azithromycin and ciprofloxacin decreased to zero, suggesting that inappropriate usage of second or third-line antibiotics was curtailed. Similarly, the number of prescriptions bearing investigations did not change significantly. We did not analyze the latter objectively as we had fewer data points (only weekly data was taken) and there was no significant decline in the absolute number of investigation requests. We observed relative improvement across all spheres of outpatient clinical care, including a relative increase in confidence in diagnosing and treating pediatric patients.

Our intervention was comparatively more basic, and the assessment tools used were online testing only. The protocol included indications for referral to the pediatrician. However, our assessment of outcome was more practical as we directly analyzed the prescription handed over to the patient as our primary outcome measure. The former studies were more focused on improving the accuracy of inpatient dosing and formulations and had a large number of dropouts and voluntary participation, leading to bias. On the other hand, in our study, we ensured that all interns and JR were mandatorily enrolled in the program [4,8].

The findings of our study were comparable to the results of a similar study done by Foster ME et al. This study compared the weekly records of a limited period of the emergency department of a tertiary hospital for medication errors and compared them with adverse drug events. They applied a resident-focused program for pharmacy-related metrics, which led to an improvement in prescription errors. We compared our data to this study because it was also done in a pre-computerized era. However, our setting, being outpatient, is more controlled, and we cater to a different population, i.e., children, where weight-based dosage plays a significant role. This study did not reveal exact teaching schedules or the contents of literature provided to the residents [19].

Another study that looked at the improvement of pediatric prescription skills after an e-learning intervention can also be compared to our study [18]. They created a short, low-cost e-learning intervention for residents. That was self-interactive and consisted of audio-visual learning as well as self-assessment tools. They showed significant improvement at three months in terms of assessment scores and prescribing skills.

The present implementation research using the PDSA cycle demonstrated a reduction in medication errors over six weeks. The parameters that we analyzed included prescription errors and objective parameters of prescription of investigations or antibiotic usage, but we also assessed subjective parameters like confidence building and receptiveness amongst the trainees. The latter parameters showed that the program was well-accepted by the trainees as well.

The strengths of this study included the implementation of computerized-based testing to assess the competence and accrual of knowledge of primary care physicians and trainees for improving prescription practice. The same could be repeated periodically to improve long-term sustenance in a semi-urban setting in Haryana. Sustenance of changes was ensured by preparing and disbursing electronic copies of formulary and protocols that could be periodically updated. Inclusion of the last batch of interns posted from October to December who were on the verge of completing their internship and thus being eligible to prescribe independently gives better generalizability.

Our study had a few limitations. Although prescription errors account for the majority of medication errors and perhaps the most vulnerable part in an outpatient setting [4], we cannot ignore the other components of medication errors, which include inscription errors, errors in dispensing, and errors in administration. Another limitation of the study was that prescriptions were assessed for error by a single pediatrician who was a part of the study. Therefore, measurement bias cannot be ruled out. No formal pedagogical techniques were used in the training of interns and JR or in designing the STP manual. However, it was ensured that the STPs confirmed the locally approved guidelines and protocols, which could vary in different contexts depending on the availability of drugs. The length of our study could not be longer because of the academic limitations. The lead pediatrician is posted for a limited period of time, but this rotation would not affect the sustenance phase.

Our study highlighted that simple measures like standardized drug dosage formulary and standard treatment protocols can be easily adapted to local settings and can improve the quality of care provided to pediatric patients. Similar techniques could be applied in primary practice or in the training of family physicians. We propose that the model used in our study for quality improvement can be adapted to reduce prescriptions at the primary and secondary care levels.

Such programs can also apply to other specialty services. To conclude, the application of standardized drug formularies and STP with a plan for sustenance can help in reducing prescription errors done by trainees, especially in a pediatric outpatient setting. Future research should address other components of prescription errors and tackle errors done at primary care centers in relevance to pediatric cases.

## Conclusions

The application of standardized drug formularies and standard treatment protocols (STPs) can help reduce prescription errors in a pediatric outpatient department, especially in a primary care setting. Expansion of such techniques to other centers could be particularly useful. However, a more robust study doing a comparative trial would be needed to confirm the evidence.

## Appendices

Appendix 1 

**Table 4:** The drug dosage formulary for the pediatric outpatient department (OPD) at CRHSP-Ballabgarh, AIIMS includes drug doses and indications. URTI: upper respiratory tract infection; UTI: urinary tract infection; BD: twice a day; TDS: three times a day; SOS: if necessary; QDS: taken four times daily; SAM: severe acute malnutrition; URTI: upper respiratory tract infection GTCS: generalized tonic-clonic seizure; RTI: respiratory tract infection; ODAC: means once a day after meals; GERD: gastroesophageal reflux disease; NCC: neurocysticercosis; OD: once daily; ORS: oral rehydration solution

Drugs	Doses	Indications
Albendazole (Syp 200mg/5ml)	At day1 and day14 ≥2 years: 400mg, <2 years: 200mg (for NCC 15mg/kg/day BD for seven days)	Worm infestation NCC
Amoxicillin (Tab 250mg, 500mg, Syp 250mg/5ml)	20mg/kg/dose TDS URTI: 10 days, sinusitis: 14 days, LRTI: 30mg/kg/dose TDS for five days	Bacterial URTI sinusitis, Asom acute tonsillitis pneumonia, pyoderma
Amoxiclav (Tab 250+125mg)	Same as amoxicillin	Same as amoxicillin
Azithromycin (Tab 250mg, 500mg, Syp 250mg/5ml)	Pertussis/enteric fever: 10mg/kg OD for five days, other illness: 10mg/kg OD on day one followed by 5mg/kg for four days	Pertussis enteric fever third-line drug for tonsillitis, RTI
Budesonide (MDI 200mcg/puff Nebulzn: 2.5mg/ml)	2-4 puff BD in asthma (minimum nebulization dose 2.5mg)	Asthma, Walri
Bromhex+Salbuta Syp 2mg Salbutamol/5ml	0.1mg/kg/dose of salbutamol TDS	Mild to moderate wheeze (Walri)
Calcium (Tab 500mg, Syp 250mg/5ml) (Syp Shelcal)	80mg/kg/day	Calcipenic rickets
Cefadroxil (Syp 250mg/5ml)	15mg/kg/BD	Bacterial urti sinusitis, Asom pneumonia, Pyoderma
Cefixime (Tab 200mg, Syp 50mg/5ml)	UTI/dysentery: 10mg/kg/day BD seven days, enteric fever: 20mg/kg/day BD 10-14 days	UTI/dysentery enteric fever
Cetirizine (Syp 5mg/5ml)	6m-2years=2.5ml od, 2-6 years=2.5ml BD, >6 years=10ml HS/5ml BD	All URTI cases do not use along with anti-histaminic cough syrup
Chlorpheniramine (Syp 2mg/5ml) (Mextra/Grilinctus)	0.1mg/kg TDS for 5-7 days (max 4mg/dose 4-6 hourly)	URTI with wet cough (all cough syrup doses according to antihistaminic content)
Ciprofloxacin (eye drops)	2 drops 6 hourly	Bacterial conjunctivitis ophthalmia neonatorum
Domperidone (Syp 1mg/ml)	0.1mg/kg/dose 8 hourly	Gerd only (not in diarrhea)
Diclofenac (Tab 50mg)	0.25-1.5mg/kg/dose to be given	Inflammatory arthritis abscess (use sparingly)
Dicyclomine (Tab 10mg, drops 10, 50mg/5ml) (Buscopan, Colicaid)	Colicaid drops 5 drops < 6 months, 6-12 months 10 drops, >12 months 15 drops SOS	Infantile colic or any colicky pain
Fluconazole (Tab 50mg,150mg)	3-5mg/kg/day for seven days for disseminated fungal infection	Disseminated fungal (skin) infection
Foracort (MDI- Formeterol 200+Budesonide 400/puff)	1-2 puff BD	Maintenance dose for asthma
Folic Acid	<6 months: 25-35mcg/day, 6m-3yrs: 50mcg/day, 4-6 yrs: 75mcg/day, 7-10: 100mcg/day, 11-14 yrs: 150 mcg/day	Anemia during phenytoin TX
Ibuprofen (Tab 200mg)	5-10mg/kg/dose 6-8 hourly	Inflammatory arthritis
Ivermectin (Tab 3mg/6mg)	0.2mg/kg single dose (can repeat after two weeks)	Drug resistant scabies, lice, filariasis
IFA (Tab 100+0.5, Syp 100mg/5ml)	3mg/kg (prophylaxis for preterm baby till one year of age), anemia 6mg/kg	Don’t prescribe during acute illness
Lactulose (Syrup)	1ml/kg/day BD/TDS for at least three months (can be extended up to one year)	Constipation (titrate upwards as per symptoms up to 30ml TDS)
Levocetirizine	2-6 years: 0.1-0.5mg/kg/dose, >6 years: 2.5mg OD/BD	All URTI
Omeprazole (Cap 20mg)/Lansoprazole (Tab 15, 30mg)	0.7mg/kg ODAC for at least 2 weeks, Lanso 0.5mg/kg/day, 1-11 years: 15mg OD, >11 years: 30mg OD (max dose 60mg)	Functional dyspepsia (Lanzol junior 15mg tablet)
ORS	10ml/kg/loose stool for replacement, 75ml/kg in 4 hours: some dehydration, 10ml/kg/hr for dehydration in SAM	Emetogenic, so, to be administered slowly
Ondansetron (Emeset)	0.15mg/kg/dose on SOS	Symptomatic relief in emesis (cancer, gastritis induced)
Metronidazole (Tab 400mg, Syp 200mg/5ml)	Amebiasis: 35-50mg/kg/day TDS 10 D, anaerobic infection: 20mg/kg/day QDS	Amebiasis anaerobic infection antibiotic also diarrhea
Paracetamol (Tab 250, 500mg, Syp 125mg/ 5ml)	15mg/kg/dose SOS or QDS	Any type of fever or pain
Phenytoin (Tab 100mg)	Initial: 5mg/kg/day BD/TDS, Maintenance: 5-8mg/kg/day OD/BD	(Start sparingly) seizure disorder
Ranitidine (Tab 50mg, 150mg, Syp 75mg/5ml)	2-4mg/kg/day BD, for GERD 4-8mg/kg	Use is limited to infant
Salbutamol (MDI 200 mcg/puff, Syp 2mg/5ml)	Syp: 0.1-0.4mg/kg/dose TDS, oral MDI: 1-2 puffs QDS	Acute asthma or Walri (with child-size spacer for all children, mask <3 yr)
Sodium Valproate (Tab 200, 400mg Syp 200mg/5ml)	20mg/kg/day BD/TDS	GTCS, absence attack myoclonic frequent febrile seizure
Tab Vit-B complex	OD	Malnutrition
Vit-D (Cap 60k IU, Syp 400 IU/ml)	Normal baby: 400 IU/day, for SAM/preterm: 800 IU/day, rickets 60k/week for six months	All children < 1 year SAM, preterm, rickets
Vit-A (Cap 25k IU/16 drops)	3000 - 4000 IU/day	Prophylaxis: At risk or with measles, malaria, HIV, diarrhea RX: xerophthalmic state
Vit-E (Cap Evion 100IU/200IU/400IU)	1 IU/kg/day sickle cell anemia: 450 IU/day, Beta-thal: 750 IU/day	Prevention of anemia of prematurity sickle cell anemia beta-thalassemia
Xylometazoline Nasal Drops (Otrivin 0.1%)	2-3 drops into each nostril up to three times daily	Sinusitis (only for a maximum of five days)
Zinc (Tab 20mg, Syp 20mg/5ml)	<6 months: 10mg/day for 14 days, >6 months: 20mg/day for 14 days, SAM: 2mg/kg/day	Diarrhoea <5years Sam
